# Large Room-Temperature Electrocaloric Effect in Lead-Free Relaxor Ferroelectric Ceramics with Wide Operation Temperature Range

**DOI:** 10.3390/ma17215241

**Published:** 2024-10-28

**Authors:** Xiaobo Zhao, Zhiyong Zhou, Bo Liang, Shengguo Lu

**Affiliations:** 1 School of Integrated Circuits, Guangdong University of Technology, Guangzhou 510006, China; 2 Guangdong Provincial Research Center on Smart Materials and Energy Conversion Devices, Guangdong Provincial Key Laboratory of Functional Soft Condensed Matter, School of Materials and Energy, Guangdong University of Technology, Guangzhou 510006, China; 3 Key Laboratory of Inorganic Functional Materials and Devices, Shanghai Institute of Ceramics, Chinese Academy of Sciences, Shanghai 200050, China

**Keywords:** electrocaloric effect, lead-free, relaxor ferroelectrics, wide operation temperature range

## Abstract

In order to obtain large room-temperature electrocaloric effect (ECE) and wide operation temperature range simultaneously in lead-free ceramics, we proposed designing a relaxor ferroelectric with a *T*_m_ (the temperature at which the maximum dielectric permittivity is achieved) near-room temperature and glass addition. Based on this strategy, we designed and fabricated lead-free 0.76NaNbO_3_–0.24BaTiO_3_ (NN-24BT) ceramics with 1wt.% BaO–B_2_O_3_–SiO_2_ glass addition, which showed distinct relaxor ferroelectric characteristics with strongly diffused phase transition and a *T*_m_ near-room temperature. Based on a direct measurement method, a large Δ*T* (adiabatic temperature change) of 1.3 K was obtained at room temperature under a high field of 11.0 kV mm^−1^. Additionally, large ECE can be maintained (>0.6 K@6.1 kV mm^−1^) over a broad temperature range from 23 °C to 69 °C. Moreover, the ECE displayed excellent cyclic stability with a variation in Δ*T* below ±7% within 100 test cycles. The comprehensive ECE performance is significantly better than other lead-free ceramics. Our work provides a general and effective approach to designing lead-free, high-performance ECE ceramics, and the approach possesses the potential to be utilized to improve the ECE performance of other lead-free ferroelectric ceramic systems.

## 1. Introduction

Ferroelectrics have potential application value in fields such as actuators, infrared detectors, non-volatile memory, and novel solid-state refrigeration [1,2]. In particular, the discovery of giant electrocaloric effect (ECE) in Pb(Zr_0.95_Ti_0.05_)O_3_ ferroelectric thin films and PVDF-based ferroelectric polymers has made it possible to design new solid-state refrigeration devices based on ECE [3,4]. The ECE refers to the adiabatic temperature change (Δ*T*) or isothermal entropy change (Δ*S*) of ferroelectrics when an external electric field is applied or removed, which is mainly caused by the change in the order of dipoles. Cooling technology based on ECE has prominent advantages such as high efficiency and environmental friendliness and is expected to replace traditional refrigeration technology based on vapor compression [5].

So far, the ECE has been observed in ferroelectric bulk ceramics, single crystals, polymers (or nanocomposite polymers), and thin films [3,4,6,7,8]. Among them, polymers and thin films exhibit the largest ECE, but the cooling capacity is very small and difficult to be directly utilized by refrigeration devices [3,4]. Additionally, ferroelectric bulk ceramics not only have large thermal mass to provide sufficient cooling capacity but also have advantages such as low cost and ease of mass production, demonstrating a very good application prospect in the field of ECE cooling. Although lead-based ferroelectric bulk ceramics exhibit excellent ECE performance, the highly toxic nature of lead limits their application scope [9,10]. Therefore, it is of great significance to investigate and develop lead-free ferroelectric bulk ceramics with high ECE performance.

Although the ECE of lead-free ferroelectric bulk ceramics such as (K, Na)NbO_3_-based, (Bi, Na)TiO_3_-based, and BaTiO_3_-based systems have been extensively studied [6,11,12,13,14], there are still several issues that have not been well addressed. Firstly, for most lead-free ceramics, the obtained Δ*T* value is still relatively small, mainly caused by the low breakdown strength. Secondly, it is difficult to simultaneously coordinate the balance between improving the ECE and expanding the operation temperature range. The improvement of ECE always comes at the cost of sacrificing the operation temperature range and vice versa.

The magnitude of ECE is closely related to the applied electric field; thus, the improvement in the breakdown strength plays a key role in enhancing the achievable ECE. There are many factors that affect the breakdown strength, including defects, porosity, density, grain size, sample thickness, test temperature, and so on [15]. Generally, the breakdown strength can be optimized by doping or special preparation process [15,16,17]. Glass addition is another effective approach to improve the breakdown strength owing to the realization of ideal density with little porosity or lower sintering temperature, which has been widely applied in the design of high-performance electrostatic energy storage materials [18,19]. On the other hand, the ECE reaches maximum around the phase transition temperature [3,4,7,8]. Consequently, due to the diffused phase transition, relaxor ferroelectric ceramics possess the potential to achieve a wide operation temperature range accompanied by large ECE.

**Figure 1:**
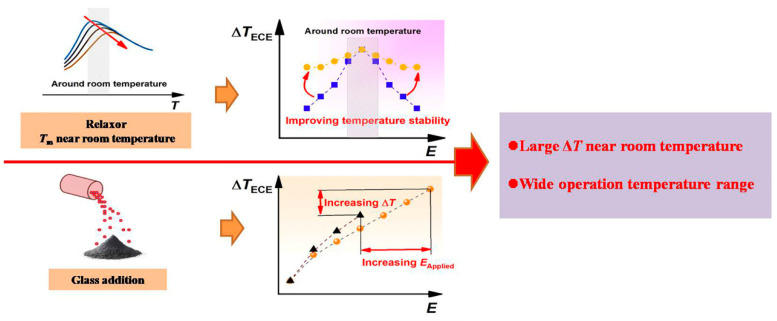
Design strategy for achieving both large Δ*T* and wide operation temperature range.

As the glass addition can improve the breakdown strength, and the relaxor ferroelectrics possess diffused phase transition, the combination of these two strategies can design high-performance lead-free ceramics with both large ECE and a wide operation temperature range. Moreover, tuning the phase transition temperature (*T*_m_) to room temperature can further achieve a large room-temperature ECE. The related design idea is illustrated in Figure 1. Based on the design idea, we designed and fabricated 0.76NaNbO_3_–0.24BaTiO_3_ (NN-24BT) lead-free ceramics, which exhibited remarkable relaxor ferroelectric characteristics with a *T*_m_ near-room temperature, and 1 wt.% BaO–B_2_O_3_–SiO_2_ glass was added to the system to increase the breakdown strength in this work. The phase structure, microstructure, dielectric properties, ferroelectric properties, and ECE of the ceramics were systematically investigated.

## 2. Experimental

**Figure 2:**
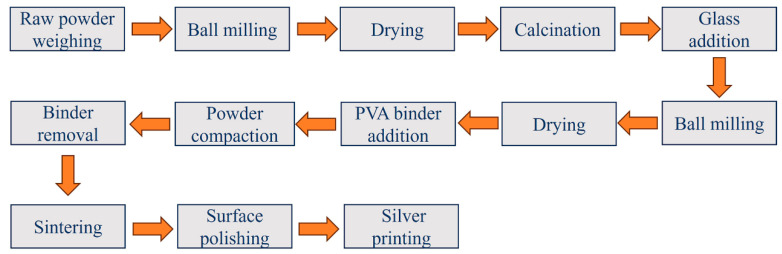
Preparation steps for the ceramic samples.

Based on the solid-state method, the NN-24BT ceramics were successfully prepared with Na_2_CO_3_ (99.5%), Nb_2_O_5_ (99.9%), BaTiO_3_ (99.5%), and TiO_2_ (99%) raw powders. According to the chemical stoichiometric ratio, the powders were weighed. The weighed powders were placed in a polyethylene ball mill jar with ZrO_2_ balls and ball-milled for 24 h. After drying, the mixture was calcinated at 900 °C for 3 h. The BaO–B_2_O_3_–SiO_2_ glass was fabricated by mixing analytical reagent grade BaCO_3_, HBO_2_, and SiO_2_ raw materials, and the mixture was melted at 1300 °C. Then, 1 wt.% BaO–B_2_O_3_–SiO_2_ glass was added to the calcinated powders. The mixed powders were ball-milled again, dried, and then mixed with 3% PVA as the binder to formulate granules. After heat treatment at 600 °C for 2 h to remove the binder, the temperature was increased to 1230 °C and held for 4 h, then cooled in the furnace. The surface of the ceramic samples was polished with sandpaper and silver-printed. The sample preparation steps are illustrated in Figure 2.

The phase structure was analyzed using an X-ray diffractometer (XRD, D8-Advance, Bruker Company, Bremen, Germany). The grain structure and densification of the ceramics were observed using a scanning electron microscope (SEM, FEI NanoSEM 630, FEI Company, Eindhoven, The Netherlands). The dielectric permittivity (*ε*) was measured using a temperature-dependent dielectric spectrometer with (E4980A). The dielectric permittivity was measured from −50 °C to 200 °C, and the measurement frequency included 100 Hz, 1 kHz, 10 kHz, and 100 kHz in this work. The ECE was directly measured by a specially designed calorimeter (see Figure S1) with a heat flux sensor.

## 3. Results and Discussion

**Figure 3:**
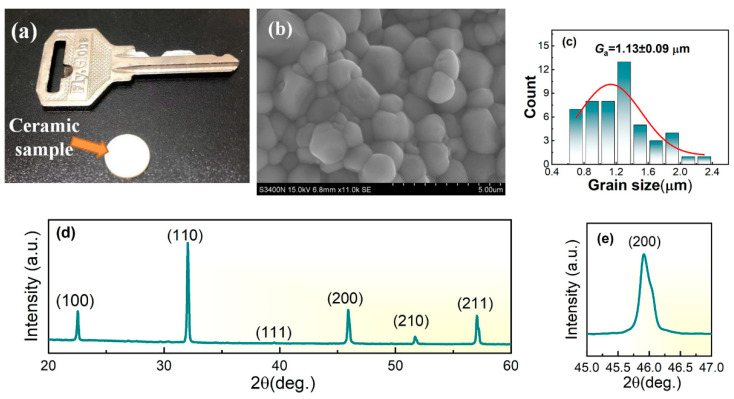
(**a**) Photograph of the sintered ceramic sample; (**b**) Surface SEM image of the sample; (**c**) Grain size distribution measured by the Image J software; (**d**) Room-temperature XRD pattern of the sample from 20° to 60°; (**e**) Enlarged image of the XRD (200) peak.

Figure 3a shows the sintered ceramic sample 10 mm in diameter. The surface SEM image of the sample is presented in Figure 3b. A dense microstructure with minimal porosity is observed, which demonstrates that a high-quality ceramic sample is fabricated. Additionally, the grain size is measured by the ImageJ2 software based on the SEM image, and the measured results are displayed in Figure 3c. The grain size distribution is fitted by the Gauss Function, and the obtained average grain size *G*_a_ is as small as 1.13 ± 0.09 μm. As the electrical resistivity of grain boundary is much higher than that of grain, the small grain size with high grain boundary density could contribute to the increase in the breakdown strength and result in the enhancement of ECE [15,17,20]. The room-temperature XRD pattern of the sample from 20° to 60° is shown in Figure 3d. A typical perovskite structure without an obvious secondary phase is formed for the sample. Moreover, a cubic phase structure is demonstrated for the sample.

**Figure 4:**
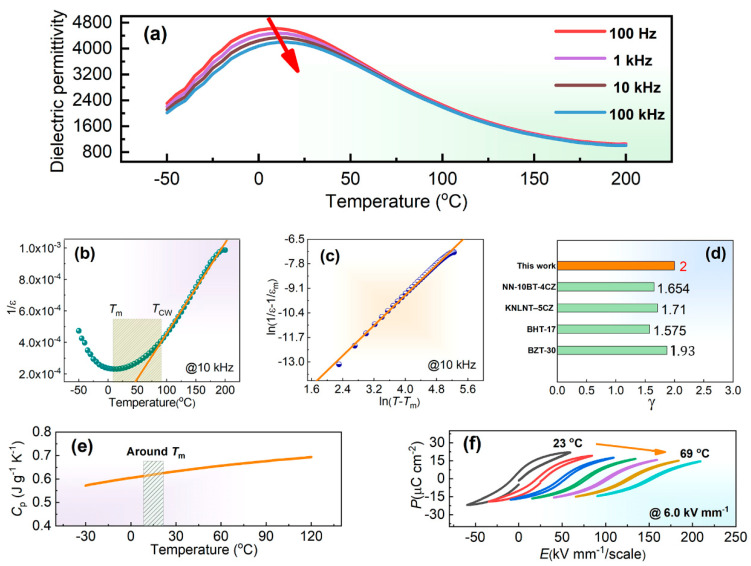
(**a**) Temperature dependence of dielectric permittivity from −50 °C to 200 °C measured at various frequencies; (**b**) 1/*ε* versus temperature curve at 10 kHz; (**c**) ln(1/*ε*−1/*ε_m_*) data as a function of ln(*T*−*T*_m_) at 10 kHz; (**d**) *γ* values obtained in the ceramic sample and other typical lead-free relaxor ferroelectric system [14,23,24,25]; (**e**) Temperature dependence of specific heat capacity *C*_p_ ranging from −30 °C to 120 °C; (**f**) *P*–*E* loops measured at various test temperatures ranging from 23 °C to 69 °C under a fixed test field of 6.0 kV mm^−1^ at 10 Hz.

Figure 4a presents the temperature dependence of dielectric permittivity from −50 °C to 200 °C measured at various frequencies. It can be seen that the maximum dielectric permittivity *ε*_m_ tends to decrease, and the *T*_m_ corresponding to the *ε*_m_ tends to increase with the increment in frequency, indicating a dielectric dispersion phenomenon, as marked by the red arrow. It needs to be noted that the dielectric dispersion phenomenon is a typical feature of the relaxor ferroelectric [14,21]. Generally, the dielectric permittivity of a normal ferroelectric above the Curie temperature obeys the following Curie–Weiss law [14,22]:

(1) $$\frac{1}{\varepsilon }=\frac{T-T_{C}}{C}(T>T_{C})\;$$

where *ε*, *T*, *T*_C_, and *C* are the dielectric permittivity, temperature, Curie temperature, and Curie–Weiss constant, respectively. Figure 4b shows 1/ε versus the temperature curve at 10 kHz. A remarkable deviation from the Curie–Weiss law is observed above the Curie temperature, further confirming the relaxor feature [14,23]. The *T*_m_ and *T*_CW_ from which the dielectric permittivity starts to deviate from the Curie–Weiss law are 10 °C and 91 °C, respectively. In addition, the *T*_CW_ is also regarded as the Burns temperature. A modified Curie–Weiss law has been proposed to estimate the diffuseness of a phase transition [14], as follows:

(2) $$\frac{1}{\varepsilon }-\frac{1}{\varepsilon_{m}}=\frac{{\left(T-T_{m}\right)}^{\gamma }}{C^{\prime }}$$

where *γ* and *C*′ are constant. The γ value reflects the phase transition characteristic, and 1 ≤ *γ* ≤ 2. *γ* = 1 denotes a normal ferroelectric, and γ = 2 indicates a completely diffused phase transition for an ideal relaxor ferroelectric [14,23]. Figure 4c shows the ln(1/*ε*−1/*ε*_m_) data as a function of ln(*T*−*T*_m_) at 10 kHz. According to the linear fitting result for the data, the γ is estimated to be 2.0, demonstrating an entirely diffused phase transition, which is rarely discovered in lead-free relaxor ferroelectrics. According to Figure 4d, the γ value is higher than that of most other typical lead-free relaxor ferroelectric systems. Figure 4e reveals the temperature dependence of specific heat capacity *C*_p_ ranging from −30 °C to 120 °C. No obvious *C*_p_ peak can be observed around the *T*_m_, which further confirms the strongly diffused phase transition [14]. The polarization–electric field (*P*−*E*) loops measured at various test temperatures ranging from 23 °C to 69 °C under a fixed test field of 6.0 kV mm^−1^ at 10 Hz are presented in Figure 4f. The *P*–*E* loop becomes slimmer, and the maximum polarization tends to decrease from 22.0 μC cm^−2^ to 14.6 μC cm^−2^ with the increase in the test temperature, which is a common phenomenon for the relaxor ferroelectric around the *T*_m_ [14].

**Figure 5:**
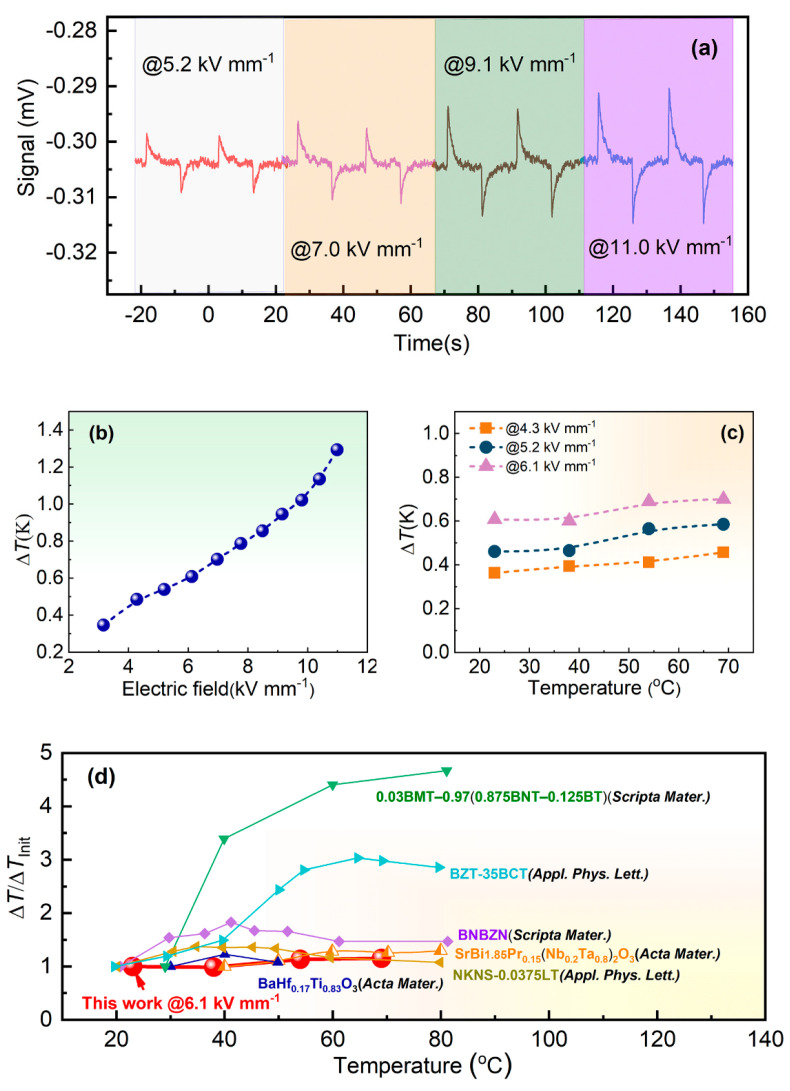
(**a**) ECE heat flow signals detected by a heat flux sensor under various electric fields at room temperature; (**b**) Room–temperature Δ*T* of ECE as a function of the test electric field; (**c**) Δ*T* value as a function of the test temperature ranging from 23 °C to 69 °C measured at various electric fields; (**d**) Comparison of the thermal stability of ECE (Δ*T*/Δ*T*_Init_) near-room temperature (20–80 °C) among the sample and other typical Lead–free ceramic systems [13,14,20,27,32,34].

The ECE heat flow signals detected by a heat flux sensor under various electric fields of 5.2 kV mm^−1^, 7.0 kV mm^−1^, 9.1 kV mm^−1^, and 11.0 kV mm^−1^ at room temperature of 23 °C are presented in Figure 5a. The exothermic peaks and endothermic peaks are induced as the external electric fields are applied and removed, respectively. The endothermic peaks are utilized to calculate the adiabatic temperature change Δ*T* of ECE in this work. It can be observed that the ECE heat flow signal curves go back to the baseline for all the test electric fields when equilibrium is reached, indicating that the Joule heat can be neglected during the test process. Figure 5b reveals the room-temperature Δ*T* of ECE for our sample as a function of the test electric field. Significant enhancement in the Δ*T* is demonstrated with the increase in the electric field. A maximum Δ*T* of 1.3 K is achieved under the highest electric field of 11.0 kV mm^−1^, which is significantly higher than that obtained in most lead-free ferroelectric bulk ceramics. This high Δ*T* obtained at room temperature should be attributed to three favorable factors: low *T*_m_ near-room temperature; remarkable relaxor ferroelectric feature; and high-test electric field. Generally, an excellent ECE performance can be observed near the *T*_m_ accompanied by a polar–nonpolar phase transition due to the easy polarization extension and rotation. Because of the remarkable relaxor ferroelectric feature confirmed by the above dielectric analysis, dynamically disordered polar nanoregions (PNRs) can be induced in the nonpolar matrix below the Burns temperature [12,14]. The PNRs accompanied by polarization can be easily switched by an external electric field due to a flat energy landscape, thus resulting in large entropy change and ECE enhancement [12,14,20]. Additionally, the high-test electric field for the ceramic sample should be ascribed to the fine grain size with high grain boundary density and dense microstructure regulated by the addition of glass [18,19]. In fact, the breakdown strength, which directly determines the highest test electric field, is increased by 45% from 8.65 kV mm^−1^ to 12.54 kV mm^−1^ (see Figures S2 and S3) with the addition of glass. Actually, the addition of BaO–B_2_O_3_–SiO_2_ glass is conducive to reducing porosity and defects accompanied by dense and uniform microstructure for the ceramic samples, thus resulting in the improvement of the breakdown strength. Moreover, the added glass can act as a sintering aid, which is beneficial to obtaining a small grain size. Due to the high electrical resistance of grain boundary, the small grain size can contribute to the further improvement of the breakdown strength.

Table 1 summarizes the Δ*T* and corresponding test field (*E*_test_) for our sample and other representative lead-free ceramics achieved at room temperature (20–40 °C) [12,13,14,20,26,27,28,29,30,31,32]. In order to facilitate comparison, all the listed Δ*T* values in the table were obtained via direct measurement methods. It can be seen that most lead-free ceramics exhibit a low Δ*T* less than 0.5 K measured directly, as well as a low test field below 6.0 kV mm^−1^ due to the low breakdown strength around room temperature. Additionally, very few lead-free bulk ceramics can achieve a Δ*T* above 1.0 K and a test field higher than 10.0 kV mm^−1^ via direct measurement approaches around room temperature. Through comparison, it can be concluded that our sample reveals a competitive room-temperature ECE performance with a high Δ*T* of 1.3 K induced by a field of 11.0 kV mm^−1^, which is superior to that of most lead-free counterparts and comparable to the best results achieved in lead-free ceramic systems. On the other hand, the most studied lead-based ceramics for ECE cooling are PMN-PT systems, which were reported to yield a Δ*T* ranging from 0.3 K to 1.6 K, depending on the composition and test field based on direct measurement methods around room temperature [9,23]. Consequently, the room-temperature ECE performance of our sample is also comparable to that of the widely studied PMN-PT ceramics, which are considered promising material candidates for ECE cooling.

**Table 1:** Δ*T* measured directly and corresponding test field (*E*_test_) for our sample and other representative lead-free ceramics achieved around room temperature (20–40 °C).

Composition	Δ*T*(ECE) (K)	*E*_test_ (kV mm^−1^)	*T* (^o^C)	Measurement Method	Journal	Reference	Publication Year
**NN-24BT+glass**	**1.3**	**11.0**	**23**	**Direct**	**This work**	**/**	**2023**
**NN-BT-0.05BZ**	**0.27**	**3.0**	**30**	**Direct**	***J Mater. Chem. A***	[26]	**2021**
**BZT-35BCT**	**0.11**	**2.0**	**20**	**Direct**	***Appl. Phys. Lett***	[27]	**2015**
**KNN-STO**	**1.2**	**15.9**	**27**	**Direct**	***Appl. Phys. Lett***	[28]	**2015**
**NKNS-0.0357LT**	**0.21**	**2.0**	**20**	**Direct**	***Appl. Phys. Lett***	[13]	**2016**
**SBPNT**	**0.14**	**5.5**	**40**	**Direct**	***Acta Mater.***	[20]	**2017**
**BaHf_0.17_Ti_0.83_O_3_**	**0.26**	**1.0**	**30**	**Direct**	***Acta Mater.***	[14]	**2016**
**BNT-BT-0.08SBT**	**0.40**	**6.0**	**27**	**Direct**	***J. Eur. Ceram. Soc.***	[29]	**2017**
**KNN-0.05BNZ**	**0.16**	**3.5**	**30**	**Direct**	***J. Am. Ceram. Soc.***	[30]	**2019**
**BNT-0.06BT**	**0.25**	**4.0**	**24**	**Direct**	***Appl. Phys. Lett***	[12]	**2017**
**BT-0.07CZ-BS**	**0.30**	**1.5**	**40**	**Direct**	***J Mater. Chem. C***	[31]	**2021**
**BNBZN**	**0.89**	**5.0**	**30**	**Direct**	***Scripta Mater.***	[32]	**2019**

A wide operation temperature range for the ECE materials is also critical for practical device applications. Figure 5c presents the Δ*T* value as a function of the test temperature ranging from 23 °C to 69 °C measured at various electric fields. Interestingly, a slight increase in the Δ*T* is observed with the increment of the test temperature for various electric fields, which is also discovered in other relaxor ferroelectric systems [20,28,33]. This phenomenon is quite different from that observed in normal ferroelectric systems in which the Δ*T* reaches the maximum value around the *T*_m_ and tends to decrease rapidly with the further increment of the test temperature. Additionally, this phenomenon should be attributed to the continued existence of PNRs caused by the structural and chemical disorders (heterogeneous lattice strain, vacancy distribution, and cation disorder) below the Burns temperature, which can be much higher than *T*_m_ for strong relaxor ferroelectric systems [20]. Consequently, according to Figure 5c, large ECE can be maintained (>0.6 K@6.1 kV mm^−1^) over a broad temperature range from 23 °C to 69 °C, which is attractive for numerous cooling application scenarios. Additionally, Figure 5d compares the thermal stability of ECE (Δ*T*/Δ*T*_Init_) near-room temperature (20–80 °C) among the sample and other typical lead-free ceramic systems including BaTiO_3_-based, (K, Na)NbO_3_-based, (Bi, Na)TiO_3_-based and Aurivillius ferroelectric ceramics [13,14,20,27,32,34]. We can see that the change in Δ*T* is very drastic for most lead-free ceramics in the selected temperature range; for instance, the variation in Δ*T* is even over 350% for 0.03BMT-0.97(0.875BNT-0.125BT) ceramics [34]. By comparison, better thermal stability of ECE performance near-room temperature is demonstrated for our sample, which is highly desired for designing high-efficiency solid-state cooling devices.

**Figure 6:**
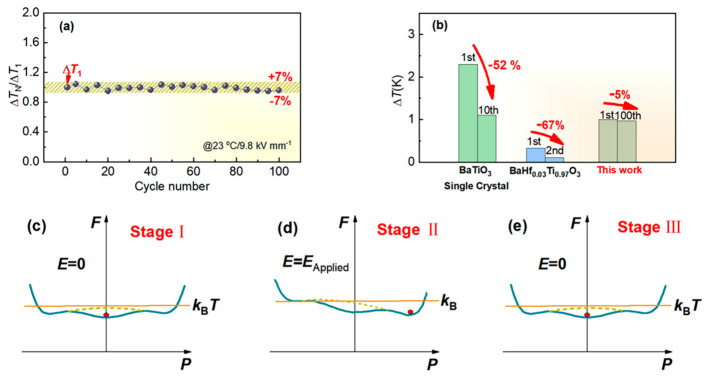
(**a**) Calculated Δ*T*_N_/Δ*T*_1_ (N denotes the Nth cycle) as a function of cycle number; (**b**) The variation in Δ*T* after ECE cycles; (**c**) Landau free energy profiles for the sample at *E* = 0 (Stage I); (**d**) Landau free energy profiles for the sample at *E* = *E*_Applied_ (Stage II); (**e**) Landau free energy profiles for the sample at *E* = 0 (Stage III).

The cyclic stability of ECE, which has been rarely explored in previous research works, needs to be focused on designing practical ECE-based cooling devices. Actually, some limited investigations implied that the test cycle might have a differential impact on ECE depending on the material type and the associated phase transition characteristic [14,35]. In order to assess the cyclic stability, 100 ECE test cycles were performed on the sample with a test field of 9.8 kV mm^−1^ at 23 °C. Figure 6a presents the calculated Δ*T*_N_/Δ*T*_1_ (N denotes the Nth cycle) as a function of cycle number, which reflects the fluctuation of ECE performance against the test cycles. Encouragingly, the variation in Δ*T* is below ±7% within 100 test cycles, indicating exceptional cyclic stability of ECE for the sample. As revealed in Figure 6b, dramatic reduction in Δ*T* was reported for some lead-free systems after several ECE cycles according to the literature, such as 52% reduction for BaTiO_3_ single crystal and 67% reduction for Ba(Hf_0.03_Ti_0.97_)O_3_ ceramics with O and T phases coexistence [14,35], while the Δ*T* for the sample decreased only by 5% after 100 ECE test cycles. In order to understand this excellent cyclic stability of ECE for the sample, a modified schematic Landau free-energy landscape, which phenomenologically describes the state of a system on the basis of both the average order parameter and the configuration coordinate, is adopted for analysis [12,14]. Generally, the free energy *F* of a ferroelectric system can be expanded into a power series of polarization (*P*), as follows [12,14]:

(3) $$\;F\left(\vec{P}\right)=F_{0}+aP^{2}+bP^{4}+cP^{6}+dP\;\;\;\;\;\;\;\;$$

where a, *b*, and *c* are the coefficients of various order terms; *d* denotes the average field induced by the external field, and *F*_0_ denotes the free energy for the paraelectric phase. Based on the above formula, the Landau free energy profiles are plotted in Figure 6c–e, in which *k*_B_*T* denotes the thermal activation energy, and the dotted line represents the local barrier that can be induced by composition or local field inhomogeneity. Due to the remarkable relaxor characteristic, a flat energy landscape and low energy barrier are demonstrated at the test temperature *T*(*T*_m_ < *T* < *T*_CW_). When *E* = 0 (Stage I), the nonpolar phase is more stable than the polar phase (see Figure 6c). When *E* = *E*_Applied_ (Stage II), the free energy of the polar phase becomes lower than that of the nonpolar phase, and the polar phase becomes more stable (see Figure 6d). At Stage III, when the electric field is removed, the system will return to the original nonpolar state due to the low energy barrier (energy barrier < *k*_B_*T*) (see Figure 6e). We believe that this reversibility in terms of free energy provides a basic insight into the cyclic stability of ECE for the sample. Moreover, minimal Joule heat generated during the test process also contributes to the cyclic stability.

## 4. Conclusions

In conclusion, based on the design strategy that the relaxor ferroelectric characteristics with a *T*_m_ (the temperature at which the maximum dielectric permittivity is achieved) near-room temperature and glass addition are realized simultaneously in lead-free ceramics, the 0.76NaNbO_3_–0.24BaTiO_3_ ceramics with 1 wt.% BaO–B_2_O_3_–SiO_2_ glass addition were designed and fabricated. According to the SEM (Scanning Electron Microscope) and XRD (X–ray Diffraction) results, the obtained ceramics exhibited dense microstructure and typical perovskite structure. The analysis of dielectric properties demonstrated that the ceramics possessed remarkable relaxor ferroelectric characteristics with strongly diffused phase transition and a *T*_m_ near room–temperature. The electrocaloric effect (ECE) was characterized by a direct measurement method. A large Δ*T* (adiabatic temperature change) of 1.3 K was obtained at room temperature under a high field of 11.0 kV mm^−1^, which was superior to that achieved in most lead-free ceramics. Additionally, large ECE can be maintained (>0.6 K@6.1 kV mm^−1^) over a broad temperature range from 23 °C to 69 °C. Moreover, the ECE displayed excellent cyclic stability with a variation in Δ*T* below ±7% within 100 test cycles. All these properties added together demonstrate that the designed, lead-free ceramics are quite promising for designing practical ECE-based cooling devices.

## Data Availability

All data generated or analysed during this study are included in this manuscript (and its supplementary information files) or can be available from the corresponding author on reasonable request.

## Supplementary Materials

The following supporting information can be downloaded at: https://www.mdpi.com/article/10.3390/ma17215241/s1, Figure S1: Specially designed calorimeter for direct ECE measurement; Figure S2: Weibull distribution of dielectric breakdown strength and related linear fittings for the NN-24BT and NN-24+Glass ceramics; Figure S3: The estimated dielectric breakdown strength according to the Weibull distribution for the NN-24BT and NN-24+Glass ceramics.
