# Supplementary material for: Large Room-Temperature Electrocaloric Effect in Lead-Free Relaxor Ferroelectric Ceramics with Wide Operation Temperature Range
Source: Materials (Basel). 2024 Oct 28;17(21):5241. doi: 10.3390/ma17215241 (PMC11547186; doi:10.3390/ma17215241)

Large room-temperature electrocaloric effect in lead-free relaxor ferroelectric ceramics with wide operation temperature range

Xiaobo Zhao[a,c*], Zhiyong Zhou[b], Bo Liang[c], and Shengguo Lu[c]

[a]School of Integrated Circuits, Guangdong University of Technology, Guangzhou, 510006, China

[b]Key Laboratory of Inorganic Functional Materials and Devices, Shanghai Institute of Ceramics, Chinese Academy of Sciences, Shanghai, 200050, Chian

[c]Guangdong Provincial Research Center on Smart Materials and Energy Conversion Devices, Guangdong Provincial Key Laboratory of Functional Soft Condensed Matter, School of Materials and Energy, Guangdong University of Technology, Guangzhou, 510006, China

Figure S1. Specially designed calorimeter for direct ECE measurement

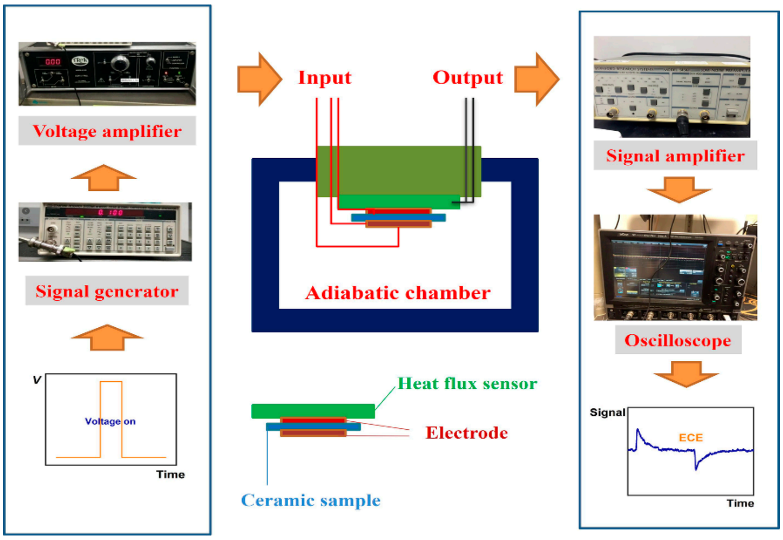

Figure S2. Weibull distribution of dielectric breakdown strength and related linear fittings for the NN-24BT and NN-24+Glass ceramics

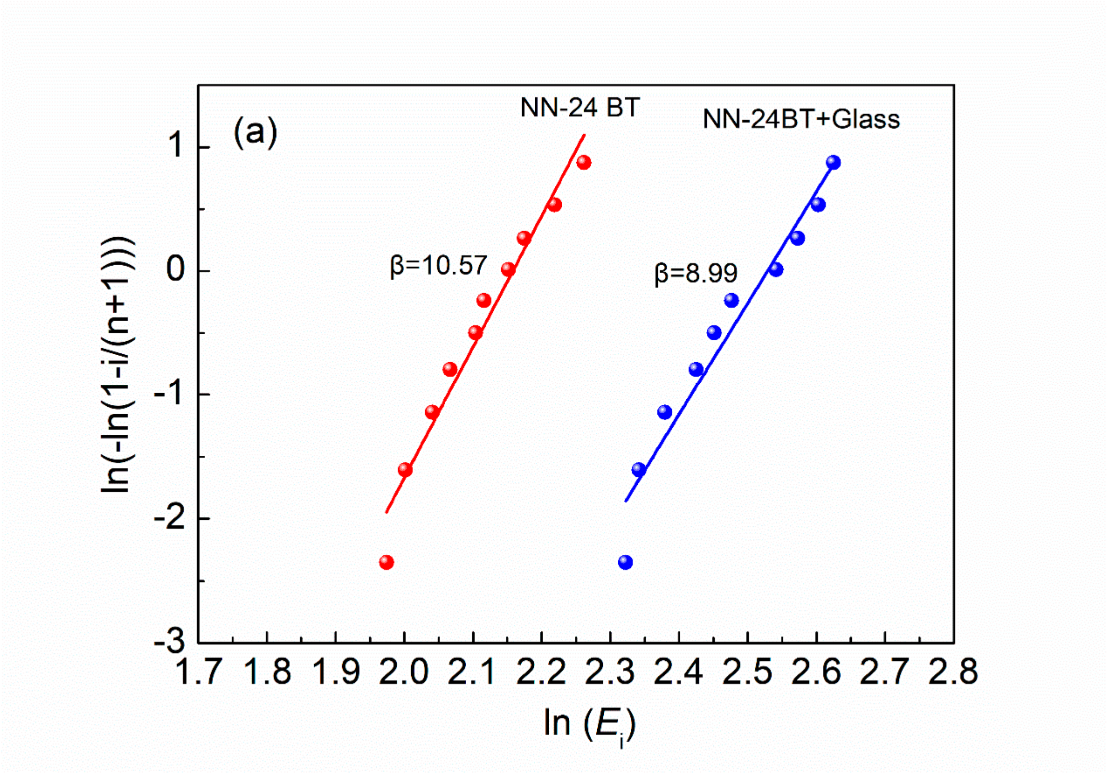

Figure S3. The estimated dielectric breakdown strength according to the Weibull distribution for the NN-24BT and NN-24+Glass ceramics

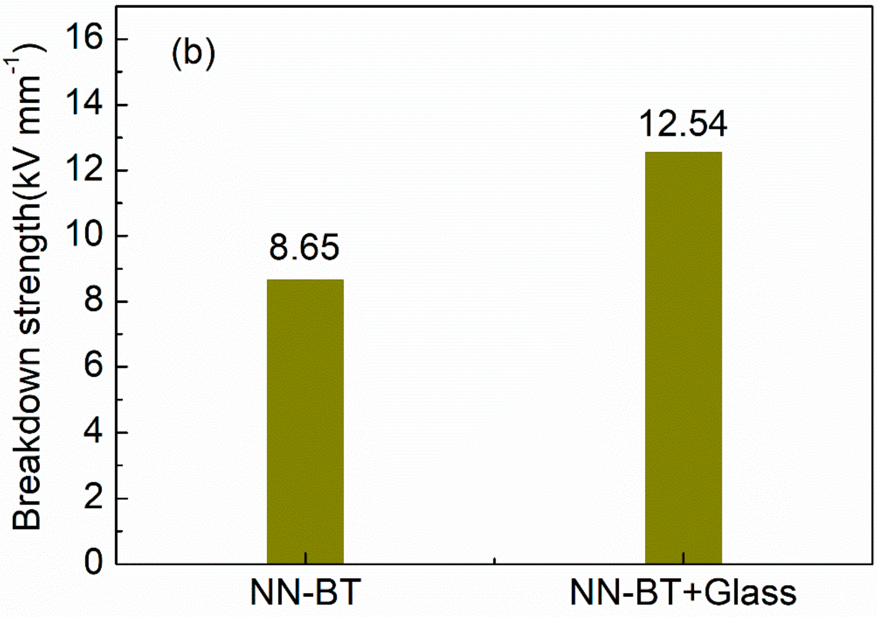

Supplement: Supplementary file 1 [file materials-17-05241-s001.zip › materials-2642508-supplementary.pdf]
